# The molecular and clinical verification of therapeutic resistance via the p38 MAPK–Hsp27 axis in lung cancer

**DOI:** 10.18632/oncotarget.7306

**Published:** 2016-02-10

**Authors:** Chia-Lin Liu, Su-Feng Chen, Min-Zu Wu, Shu-Wen Jao, Yaoh-Shiang Lin, Chin-Yuh Yang, Tsai-Yu Lee, Lian-Wu Wen, Guo-Lun Lan, Shin Nieh

**Affiliations:** ^1^ Graduate Institute of Life Sciences, National Defense Medical Center, Taipei, Taiwan; ^2^ Department of Dental Hygiene, China Medical University, Taichung, Taiwan; ^3^ Gene Expression Laboratory, Salk Institute for Biological Studies, La Jolla, California, USA; ^4^ Institute of Environmental and Occupational Health Sciences, School of Medicine & Division of Colon and Rectum Surgery, Department of Surgery, National Yang-Ming University & National Defense Medical Center, Tri-Service General Hospital Songshan Branch, Taipei, Taiwan; ^5^ Department of Otolaryngology-Head and Neck Surgery, Kaohsiung Veterans General Hospital, Kaohsiung, Taiwan; ^6^ Department of Dentistry, Cheng Hsin Hospital & Taipei Medical University, Taipei, Taiwan; ^7^ Department of Pathology, National Defense Medical Center & Tri-Service General Hospital, Taipei, Taiwan

**Keywords:** cisplatin-based chemotherapy, drug-resistant sphere, lung cancer, heat shock protein 27, treatment strategy

## Abstract

Treatment failure followed by relapse and metastasis in patients with non-small cell lung cancer is often the result of acquired resistance to cisplatin-based chemotherapy. A cancer stem cell (CSC)-mediated anti-apoptotic phenomenon is responsible for the development of drug resistance. The underlying molecular mechanism related to cisplatin resistance is still controversial, and a new strategy is needed to counteract cisplatin resistance. We used a nonadhesive culture system to generate drug-resistant spheres (DRSPs) derived from cisplatin-resistant H23 lung cancer cells. The expressions of drug-resistance genes, properties of CSCs, and markers of anti-apoptotic proteins were compared between control cells and DRSPs. DRSPs exhibited upregulation of cisplatin resistance-related genes. Gradual morphological alterations showing epithelial-to-mesenchymal transition phenomenon and increased invasion and migration abilities were seen during induction of DRSPs. Compared with control cells, DRSPs displayed increased CSC and anti-apoptotic properties, greater resistance to cisplatin, and overexpression of p-Hsp27 via activation of p38 MAPK signaling. Knockdown of Hsp27 or p38 decreased cisplatin resistance and increased apoptosis in DRSPs. Clinical studies confirmed that the expression of p-Hsp27 was closely associated with prognosis. Overexpression of p-Hsp27 was usually detected in advanced-stage patients with lung cancer and indicated short survival.

**Summary:**

DRSPs were useful for investigating drug resistance and may provide a practical model for studying the crucial role of p-Hsp27 in the p38 MAPK–Hsp27 axis in CSC-mediated cisplatin resistance. Targeting this axis using siRNA Hsp27 may provide a treatment strategy to improve prognosis and prolong survival in lung cancer patients.

## INTRODUCTION

Lung cancer is one of the leading causes of cancer-related deaths worldwide. This disease is classified as either small cell lung carcinoma or non-small cell lung carcinoma (NSCLC) [1]. An aggressive type of lung cancer, NSCLC accounts for about 85% of lung cancer and is associated with poor prognosis and a low 5-year survival rate [2]. Cancer recurrence followed by relapse and metastasis after therapy is the major cause of failure of lung cancer treatment and probably results from the resistance of tumors to chemotherapy [3]. It is thus important to elucidate the mechanisms that mediate the generation of chemoresistance in NSCLC.

Platinum-based chemotherapy is used widely for lung cancer treatment [4]. The most commonly used drug for chemotherapeutic administration in lung cancer, cisplatin, is a platinum compound that crosslinks DNA to induce apoptosis of tumor cells [5]. Cisplatin-based chemotherapy reduces the risk of death from NSCLC by about 5% and has been reported to increase the 5-year survival after complete resection by 4.1% [6]. However, previous studies of lung cancer have demonstrated the existence of cisplatin resistance [7], which may lead to treatment failure. The recent National Comprehensive Cancer Network guidelines recommend the use of cisplatin-based chemotherapy with or without targeted therapy for advanced NSCLC [8]. However, drug resistance to cisplatin-based chemotherapy inevitably develops. Despite the association of cisplatin resistance with a poor prognosis in patients with ovarian cancer, oral cancer, or lung cancer [9-11], the current understanding of the molecular mechanism remains contradictory.

The concept of cancer stem cells (CSCs) was raised decades ago based on the similarities between cancer cells and normal stem cells [12]. The existence of CSCs was first described in the context of leukemia [13]. The CSC hypothesis suggests that CSCs comprise a small population of cells among the tumor cells and possess ability for tumor formation and self-renewal [14]. Accumulating evidence suggests that CSCs can cause cancer relapse and metastasis, and can contribute to the resistance of tumors to chemotherapy [15]. Despite the link between the induction of CSCs in tumor cells, acquisition of drug resistance, and recurrence of tumors, the underlying mechanisms remain largely unknown.

Heat shock proteins (Hsps) are generally induced by environmental stress and function as molecular chaperones that are responsible for maintaining the correct conformation of other proteins. Hsps are classified into high-molecular-weight Hsps, such as Hsp90 and Hsp70, and low-molecular-weight Hsps, including Hsp27 [16]. In addition to its conventional function as a chaperone, Hsp27 has been reported to be overexpressed in breast cancer, ovarian cancer, and head and neck cancer [17-19]. The expression of Hsp27 appears to be associated with the prognosis and survival rate of patients with different cancers. Hsp27 is thought to be associated with chemoresistance and induction of tumor cells with stem cell-like properties in breast cancer and head and neck cancer. However, the involvement of Hsp27 in lung cancer is not fully understood and merits further study.

Our research team has previously established a novel nonadhesive sphere culture system that allows us to purify and enrich a population of oral squamous cell carcinoma cells with stem cell-like properties [20, 21]. Using this established technique to generate drug-resistant spheres (DRSPs), we aimed to identify the possible molecular role of phosphorylated Hsp27 (p-Hsp27) and its related signaling pathway in the control of apoptosis and drug resistance. We performed a clinicopathological study using immunohistochemical methods to study the relationships between the immunoexpression of p-Hsp27 and clinical parameters in samples from cisplatin-treated patients. Our findings may provide insight into drug resistance and ideas for new strategies against drug resistance, which may help improve the prognosis and prolong survival of patients with lung cancer.

## RESULTS

### Characterization of DR cells showing upregulation of drug-resistance genes, EMT phenomenon, and increased migration and invasion abilities

**Figure 1:**
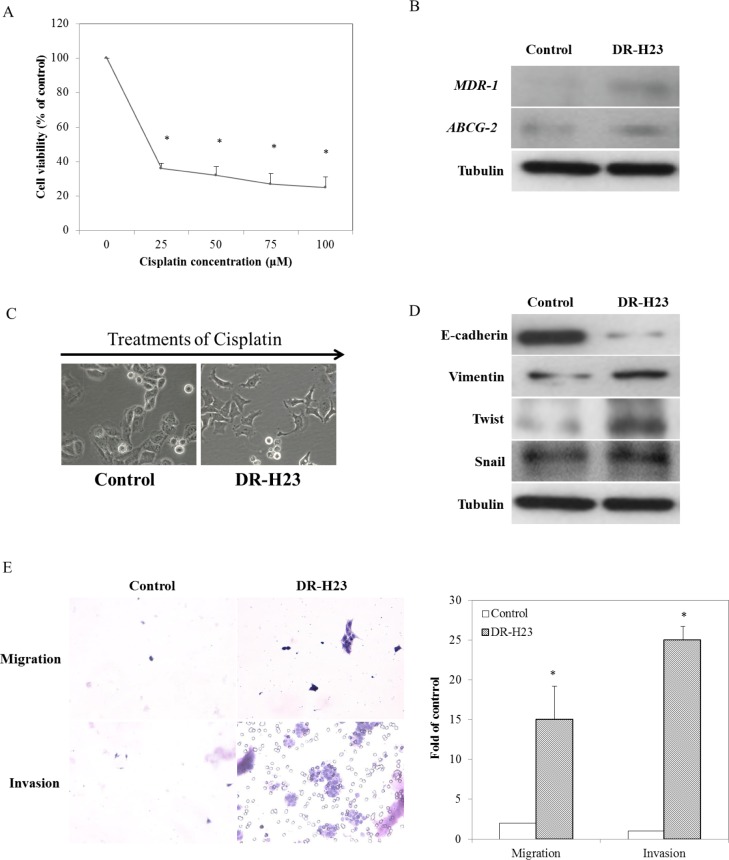
Characteristics of DR cells in terms of gene expression, the EMT phenomenon, and associated migration and invasion **A.** MTT assay demonstrated that more than 20% of all H23 cells were significantly found surviving and successfully maintained (*p* < 0.05). **B.** Western blotting showed that *ABCG-2* and *MDR-1* were upregulated in DR-H23 cells compared with control cells. **C.** An interesting phenomenon of EMT with morphological transformations was seen in DR-H23 cells. **D.** Western blotting showed alterations of EMT-representative markers, including decreased expression of E-cadherin and increased expression of vimentin, twist, and snail in DRSPs compared with control cells. **E.** Increased migration and invasion abilities were significantly evident in DR-H23 cells (*p* < 0.05).

Considering that drug resistance following chemotherapy in advanced lung cancer is a common phenomenon, we aimed to establish a model to study chemoresistance. We first established DR cells that were derived from NSCLC H23 cells by applying multistep cisplatin treatment at various concentrations (0, 25, 50, 75, and 100 μM). More than 20% of DR-H23 cells were significantly maintained after cisplatin treatment at various concentrations, as shown in an MTT assay (*P* < 0.05; Figure 1A). To study the role of DR-H23 cells in cisplatin-resistant lung cancer, we used western blotting to measure the expression of two important genes related to drug resistance, *ABCG-2* and *MDR-1* [25, 26]. The expression of these genes was upregulated significantly in DR-H23 cells compared with control cells (Figure 1B). During induction with various concentrations of cisplatin, there was a gradual transformation from an epithelioid to a mesenchymal-like cellular phenotype, suggestive of the EMT (Figure 1C). Further evaluation by western blotting showed significant alterations in EMT-representative markers, including decreased expression of E-cadherin and increased expression of vimentin, twist, and snail in DR-H23 cells compared with control cells (Figure 1D). The EMT is involved in cell mobility and progression other than morphological transformation. Therefore, migration and invasion assays were performed, and the results showed significantly increased abilities in DR-H23 cells compared with control cells (*P* < 0.05; Figure 1E).

### Demonstration and characterization of DRSPs with CSC properties

**Figure 2:**
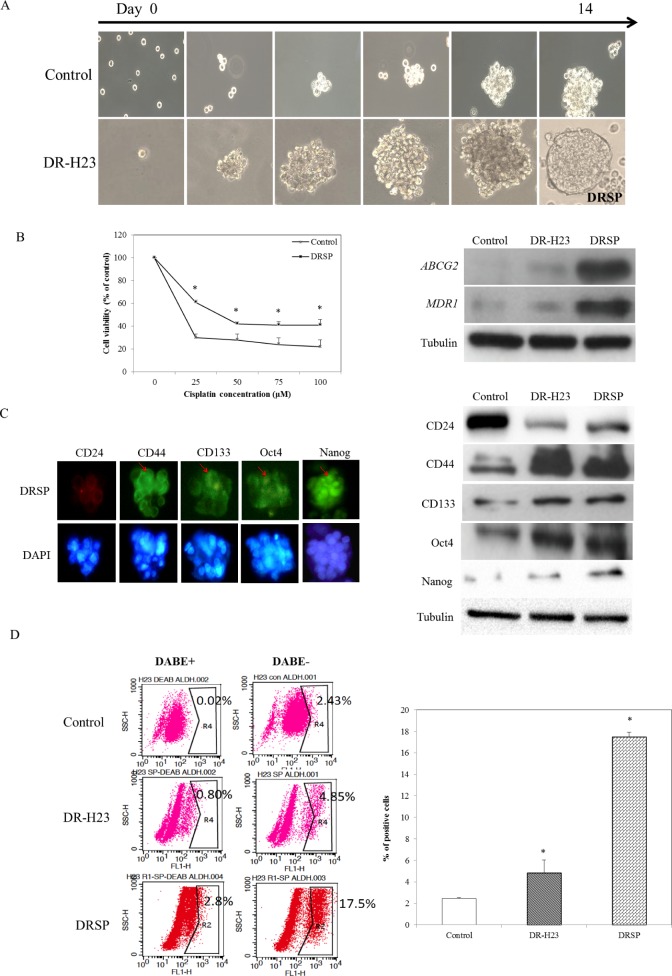
DR cells exhibiting CSC properties **A.** Using the nonadhesive sphere culture system, the derivative DR-H23 cells were cultured further for 14 days and generated a spheroid phenotype termed DR spheres (DRSPs). **B.** MTT assay showed that more surviving cells were significantly found and maintained in DRSPs than those of control cells (*p* < 0.05). Western blotting showed that the drug resistance-related genes *ABCG-2* and *MDR-1* were obviously upregulated in DRSPs compared with DR-H23 and control cells. **C.** Immunofluorescence assay and western blotting showed overexpression of CSC-representative markers, including CD44^high^/CD24^low^, CD133, Oct4, and Nanog, in DRSPs compared with DR-H23 and control cells. **D.** Flow cytometric analysis of ALDH1 showed significantly increased activity in DRSPs compared with DR-H23 and control cells (*p* < 0.05).

Increasing evidence [27-29] suggests that the EMT plays a role in both early invasion and late metastasis, and that there is a link between CSCs and the EMT. We next examined whether the CSC properties of the surviving DR-H23 cells were related to characteristics other than drug resistance and invasive capability. The derived DR-H23 cells were subjected to the nonadhesive sphere culture system and cultured for 14 days, after which they exhibited a spheroid phenotype, which we termed DRSPs (Figure 2A). An MTT assay showed larger IC_50_ values for cisplatin in DRSPs than in control cells. The results of western blotting of the related drug-resistance genes, *ABCG-2* and *MDR-1* showed that these two genes were upregulated significantly in DRSPs compared with DR-H23 and control cells (*P* < 0.05; Figure 2B). Because CSCs contribute to drug resistance in lung cancer, we aimed to elucidate the role of CSCs in the resistance of lung cancer cells to cisplatin. Immunofluorescence and western blotting assays revealed that CSC-representative markers, including CD44^high^/CD24^low^, CD133, Oct4, and Nanog, were overexpressed in DRSPs compared with DR-H23 and control cells (Figure 2C). Flow cytometry was used to analyze another important CSC-representative marker, ALDH1 [30, 31], and showed a significant increase in ALDH1 activity in DRSPs compared with DR-H23 and control cells (*P* < 0.05; Figure 2D).

### Involvement of the p38 MAPK–Hsp27 anti-apoptotic pathway in DRSPs

**Figure 3:**
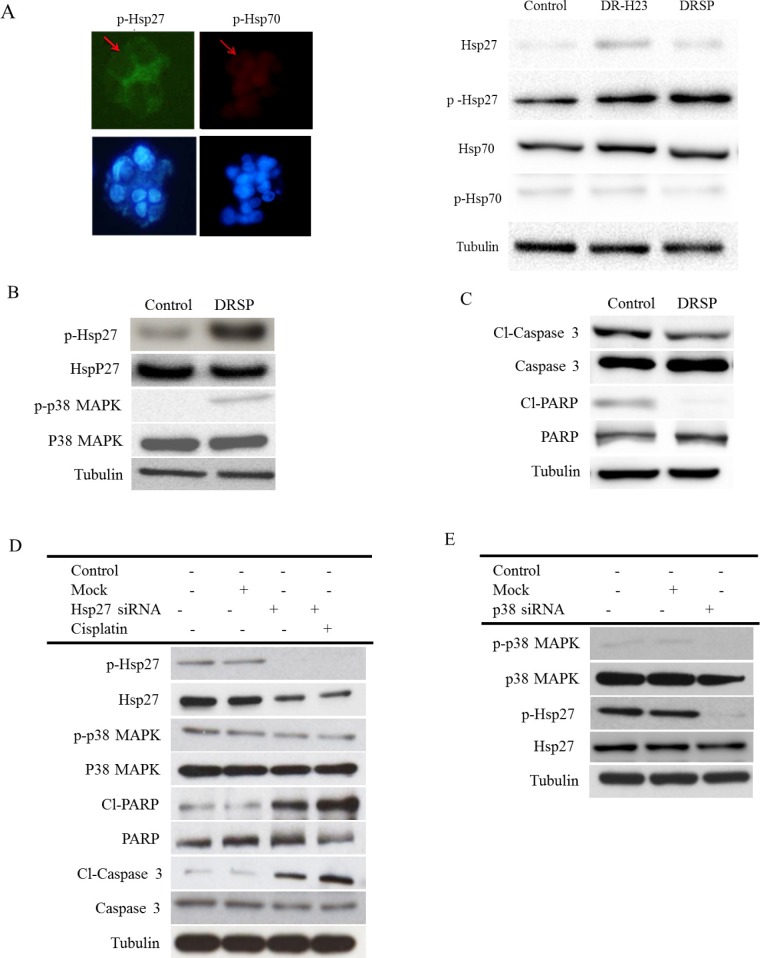
Comparison of the p38 MAPK–Hsp27 axis between control cells and DRSPs before and after knockdown of Hsp27 and P38 MAPK **A.** Immunofluorescence and western blotting assay of p-Hsp27 and p-Hsp70, two main anti-apoptotic proteins, showed that only p-Hsp27 was upregulated compared with p-Hsp70. **B.** Western blotting showed that p-Hsp27, but not Hsp27, was overexpressed via the activation of upstream p-p38 MAPK signaling in DRSPs compared with control cells. **C.** As a result, expression of the downstream-related apoptotic proteins Cl-caspase 3 and Cl-PARP was markedly decreased in DRSPs compared with control cells. **D.** SiRNA Hsp27 to increase apoptosis in DRSPs resulted in upregulation of Cl-caspase 3 and Cl-PARP, but there was no effect on p38 MAPK expression. **E.** Knockdown of p38 MAPK inhibited the expression of p-Hsp27, but not Hsp27, in DRSPs.

Because of the close relationship between chemoresistance and Hsps, especially Hsp27 and Hsp70, the two most important Hsps, we wondered which of these Hsps might be involved in our model of drug resistance and anti-apoptosis. Immunofluorescence and western blotting assays showed increased expression of p-Hsp27 compared with p-Hsp70. Western blotting showed no obvious differences in the expression of Hsp27 and Hsp70 between DRSPs and control cells (Figure 3A). Because the action of Hsp27 is initiated by p38 MAPK signaling-mediated phosphorylation [32], we measured the levels of p-Hsp27 and p38 in DRSPs. As shown in Figure 3B, the phosphorylation of Hsp27 and p38 was increased in DRSPs compared with control cells. However, expression of the downstream-related apoptotic proteins Cl-caspase 3 and Cl-PARP was markedly decreased in DRSPs (Figure 3C). Short interfering RNA (siRNA)-mediated repression of p-Hsp27 markedly reduced cisplatin resistance in DRSPs. Knockdown of Hsp27 expression induced the apoptosis of DRSPs, as shown by the activation of caspase 3 and PARP. Repression of Hsp27 did not affect the phosphorylation of p38 in DRSPs, which indicated a downstream role of Hsp27 in p38 MAPK signaling (Figure 3D). However, knockdown of p38 in DRSP cells decreased downstream p-Hsp27 expression and increased the resulting number of apoptotic events (Figure 3E). Collectively, the above results suggest that the cisplatin resistance of DRSPs results from the activation of the p38 MAPK–Hsp27 axis.

### Relationship between immunoexpression of p-Hsp27 and clinicopathological parameters in patients with lung adenocarcinoma

**Figure 4:**
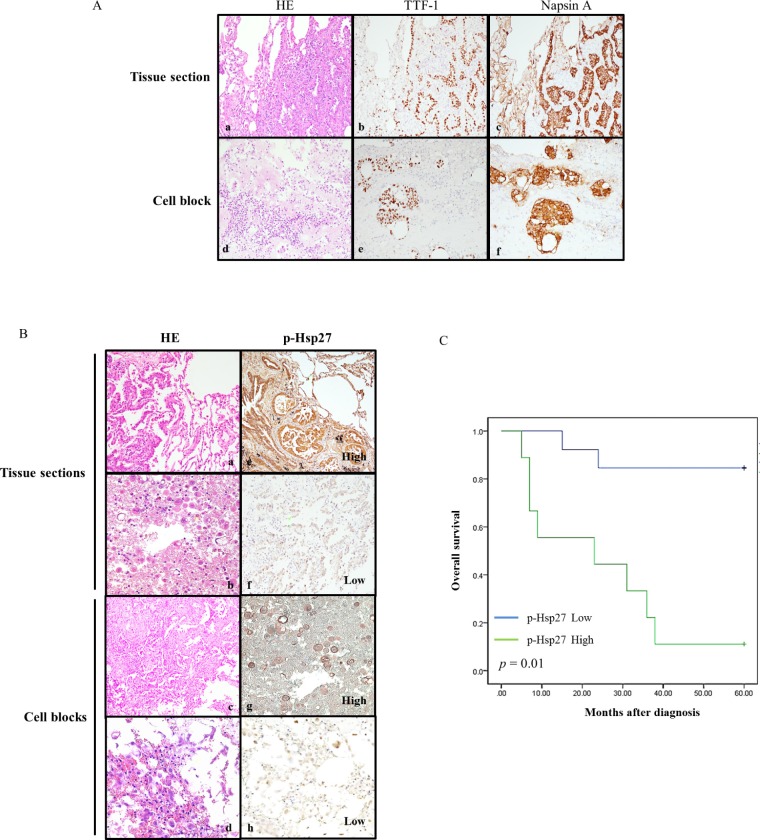
Relationships between p-Hsp27 immunoexpression in tissue sections and cell blocks, and with overall survival **A.** Histological and immunohistochemical identification of adenocarcinoma cells in tissue sections (a, b, & c) and cell blocks (d, e, & f) showing positive staining for TTF-1 and Napsin A (magnification, ×200). **B.** Histological analysis of tissue sections (a & b) and cell blocks (c & d) from four representative patients with pulmonary adenocarcinoma. The corresponding immunoexpression of p-Hsp27 is shown on the right (e, f, g, & h). The samples are from two patients whose tissues exhibited strong intensities with high immunoscores (e & g), and another two patients whose tissues exhibited weak intensities with low immunoscores (f & h) (magnification, ×200). **C.** Kaplan–Meier survival analysis of the relationship between immunoexpression of p-Hsp27 and survival after a 60-month follow-up. P-Hsp27 expression correlated significantly with overall survival (*p* = 0.01). Greater p-Hsp27 expression indicated shorter survival in patients with lung cancer.

To confirm the clinical significance of p-Hsp27 immunoexpression, we examined tissue specimens from 22 representative pulmonary adenocarcinoma patients with a history of cisplatin treatment whose detailed clinical data were available. Each individual case was reevaluated using routine hematoxylin and eosin (HE) and immunohistochemical stains for TTF-1 and Napsin A. All samples were strongly or moderately immunoreactive to TTF-1 and Napsin A, which confirmed the diagnosis of pulmonary adenocarcinoma (Figure 4A). The immunoexpression of p-Hsp27 varied according to the tumor characteristics. The expression of p-Hsp27 correlated significantly with the histological grade, tumor size, nodal involvement, metastatic status, and survival (Table 1). A strong p-Hsp27 expression with relatively high immunoscore was associated with poor prognosis in terms of nodal involvement, distant metastasis, and overall survival. By contrast, a weak intensity of p-Hsp27 expression with relatively low immunoscore was usually detected in patients with relatively low-grade cancer or non-advanced cancer (Figure 4B). Kaplan–Meier survival analysis of the relationship between the immunoexpression of p-Hsp27 and overall patient survival after a 60-month follow-up showed that p-Hsp27 expression correlated inversely with overall survival (*P* = 0.01). In other words, the stronger the p-Hsp27 expression, the shorter the overall survival in patients with lung cancer (Figure 4C).

**Table 1:** Univariate analysis for correlations between the immunoexpression of p-Hsp 27 and the clinical variables in patients with lung adenocarcinoma

Clinical Variables	p-Hsp27			*P*-value
		Low (n=14)	High (n=8)	
**Age**				
<60	6	3	3	0.389
>60	16	11	5	
**Gender**				
Male	7	5	2	0.998
Female	15	9	6	
**Differentiation**				
Well/moderately	11	10	1	<0.001**
Poorly	11	4	7	
**Tumor size (T)**				
<5cm	12	11	1	<0.001**
>5cm	10	3	7	
**Regional lymph nodes (N)**				
N0	10	9	1	0.003*
N1/N2/N3	12	5	7	
**Distant metastasis (M)**				
No	12	11	1	<0.001**
Yes	10	3	7	
**Stage**				
I/II	12	11	1	<0.001**
III/IV	10	3	7	
**Overall survival**				
Yes	12	11	1	<0.001**
No	10	3	7	

## DISCUSSION

Pulmonary adenocarcinoma, one of the most common NSCLCs, is a major cause of cancer death throughout the world. Cisplatin-based chemotherapy is used widely to improve the prognosis and survival rate after clinical treatment of advanced lung cancer, especially in patients with stage II or higher cancer. Targeted therapy to prolong the survival is regarded as another choice of treatment for stage IV patients with mutation of candidate gene(s), such as *EGFR* or *ALK* [33]. Unfortunately, cisplatin resistance is a major cause of tumor recurrence or metastasis that leads to the failure of clinical treatment and poor survival. Thus, finding new methods to address this treatment dilemma associated with drug resistance has become important in the clinical management of patients with NSCLC. Better understanding of the mechanism underlying chemoresistance is crucial to finding new treatment strategies for advanced lung cancer. A review of the literature has shown that chemoresistance during chemotherapy is ultimately controlled by CSCs within tumors [34]; in other words, CSCs are responsible for the development of resistance to chemotherapy [15]. For this purpose, we used a nonadhesive sphere culture system to isolate lung cancer cells with stem cell properties from cisplatin-resistant cells, which we term DRSPs, as previously described in oral cancer [21]. During the process of induction of DR-H23 cells from lung cancer cells using various concentrations of cisplatin, we observed the gradual morphological transformation from an epithelioid to a mesenchymal-like phenotype. This so-called EMT was initially discovered in studies of embryonic tissue migration [35, 36]. The EMT phenomenon is known to be involved in cancer progression and is closely associated with the “stemness” of cancer cells [27, 28]. As shown in Figure 1C and 1D, the isolated surviving DR cells were identified by the alterations in EMT-representative markers, including decreased expression of E-cadherin, increased expression of vimentin and two main EMT regulators, twist and snail, and significant increases in the migration and invasion abilities compared with control cells.

To study the role of “stemness”, we used a nonadhesive sphere culture system to generate DRSPs. Sphere formation indicated the capability for self-renewal, one of the most important properties of CSCs. Upregulation of the drug resistance-related genes *ABCG-2* and *MDR-1* was found in DRSPs (Figure 2B). The upregulation of these two genes that confer drug resistance suggests that the DRSPs exhibited another essential characteristic of CSCs, and provides evidence to support the hypothesis that CSCs contribute to the drug resistance of tumor cells [37]. Figure 2 shows that, in addition to the increased ALDH1 activity shown by flow cytometry, immunofluorescence and western blotting assays exhibited overexpression of the CSC-representative markers CD44^high^/CD24^low^, CD133, Oct4, and Nanog in DRSPs. Collectively, these data suggest that DRSPs exhibited the essential properties of CSCs and that these cells may provide a valid model for studying the therapeutic resistance of cancer cells. It will be interesting to further investigate the mechanisms underlying the activation of these drug-resistance genes and properties of CSCs in DRSPs and their significance in cisplatin resistance.

**Figure 5:**
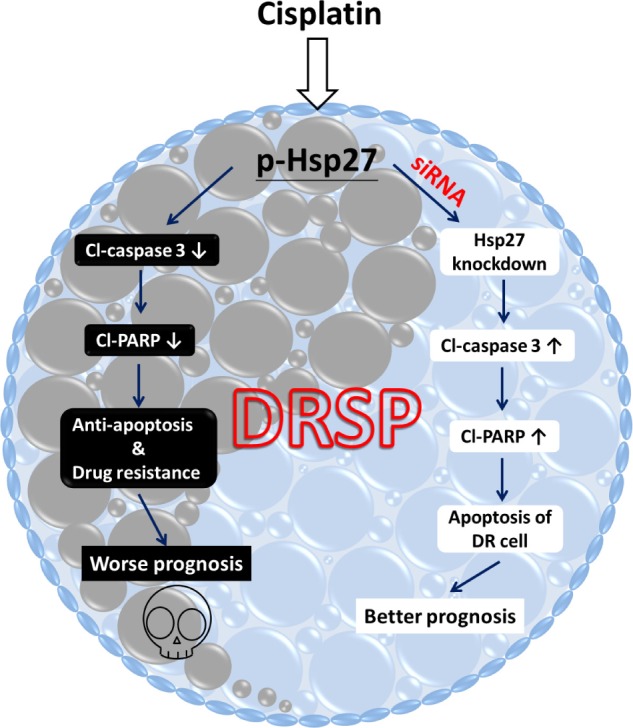
Diagrammatic illustration of the DRSP study model showing the functional role of p-Hsp27 and the p38 MAPK–Hsp27 axis in drug resistance In our DRSP study model, drug resistance of lung cancer cells following cisplatin-based chemotherapy is induced by p-Hsp27, which plays a critical role as an anti-apoptotic functional protein that inhibits the apoptotic proteins Cl-caspase 3 and Cl-PARP, via the p38 MAPK–Hsp27 axis. This ultimately leads to anti-apoptotic activity and worse prognosis in patients with lung cancer, as shown on the left. Use of siRNA Hsp27 may be a potential new treatment strategy to inhibit the p38 MAPK–Hsp27 signaling pathway, which may increase apoptosis and improve the prognosis, as shown on the right.

Hsps are generally categorized into several different families on the basis of their molecular weight. There is increasing evidence of the role of Hsp27 in drug resistance and CSC formation in breast cancer, head and neck cancer, and lung cancer [21, 38, 39]. In addition, Hsp27 function is controlled by posttranslational modifications such as phosphorylation, for which p38 MAPK signaling is responsible and which initiates Hsp27 function. Here, we found results similar to those reported previously [21, 40]. That is, phosphorylation of either Hsp27 or p38–ERK is increased in DRSPs; this suggests that the activation of p-Hsp27 is triggered by the canonical p-p38–MAPK cascade. Moreover, knockdown of Hsp27 expression in DRSPs decreased cisplatin resistance and induced apoptosis by activating caspase signaling. Taken together, these findings suggest that p-Hsp27 plays an essential role in cisplatin resistance in DRSPs. In accordance with a previous study of the role of chemoresistance in lung cancer cells [39], we propose that suppressing the function of p-Hsp27 may provide a new treatment tool for overcoming cisplatin resistance, based on its anti-apoptotic effect. A diagrammatic illustration showing our DRSP model developed to investigate the molecular mechanisms underlying drug resistance to cisplatin-based chemotherapy via p-Hsp27 is proposed in Figure 5.

Finally, to add a clinical viewpoint and to confirm the significance of p-Hsp27 expression, we analyzed samples from 22 representative cases of pulmonary adenocarcinoma patients with available clinical information. After reconfirming each individual case of pulmonary adenocarcinoma, we found that p-Hsp27 expression correlated closely with prognosis (Figure 4). This clinical significance of Hsp27 has also been suggested in two reports involving gastric cancer patients [41, 42]. Comparative analysis showed that the expression of p-Hsp27 correlated significantly with the histological grade, tumor size, nodal involvement, metastatic status, and patient survival (Table 1). A high p-Hsp27 expression level was associated with poor prognosis in terms of nodal involvement, distant metastasis, and overall survival.

In conclusion, our results provide insight into the mechanism underlying drug resistance and demonstrate that the p38 MAPK–Hsp27 axis plays an important role in the CSC-mediated cisplatin resistance of lung cancer. Targeting this axis using siRNA p-Hsp27 has potential as a treatment strategy to improve prognosis in patients with pulmonary adenocarcinoma.

## MATERIALS AND METHODS

### Cell preparation and subsequent nonadhesive sphere culture system

The human lung cancer cell line NCI-H23, was cultured in RPMI supplemented with 10% fetal bovine serum (FBS) at 37°C in the presence of 5% CO_2_. The well-established cell line was purchased from American Type Cell Collection (CRL-5800). Verification of NCI-H23 for short tandem repeat profiling was under the investigation of Academia Sinica. The last check-up test was performed in March, 2015. The cell was cultured in culture plastic wares with non-adhesive surface. 10 cm dish are made of non-adhesive for cells by coating with agarose thin films. Cells were plated at a density of 5×10^4^ live cells/10 cm dish, and the culture medium was changed every other day until the sphere formation, as seen in our previous reported [2].

### Induction of drug resistant (DR) cells

NCI-H23 parental cells were plated at a density of 5×10^4^ live cells/10 cm dish, continued treating with cisplatin were added at the final concentrations (0, 25, 50, 75, and 100 μM) for three month consequently. After treatment cells were harvested and maintaining low concentration cisplatin (0.5 μM) culture medium and the culture medium was changed every other day.

### Cell viability analysis

The cisplatin Cis was added at a dose rate of (0, 25, 50, 75, and 100 μM). Cells were seeded on 6-well plates at a density of 2 × 10^4^ per well in medium, after treatment with cisplatin for 48 h, and analyzed by the MTT assay (Sigma-Aldrich)

### RNA interference

The siRNA oligos of Hsp 27 consisted of three targets specific siRNAs designed to knockdown gene expression. The sequence of three targets were showed in below: sc-29350A: sense:5′-GAGUGGUCGCAGUGGUUAGtt-3′; antisense: 5′-CUAACCACUGCGACCACUCtt-3′); sc-29350B: sense: GACGAGCUGACGGUCAAGAtt; antisense: UCUUGACCGUCAGCUCGUCtt; sc-29350C: Sense: CCACGCAGUCCAACGAGAUtt; Antisense: AUCUCGUUGGACUGCGUGGtt or negative control siRNA oligos was purchased from Santa Cruz Biotechnologies, Inc. 2 × 10^6^ cells were transfected with a final concentration (100 nM) of Hsp 27 siRNA using Lipofectamine 2000 for 48 h to detect protein level. Cells which were transfected with non-specific siRNA (MOCK group) were paralleled demonstrated.

### Western blotting analysis

Whole cell lysates were separated by electrophoresis on 12% SDS–PAGE and transferred to polyvinylidene fluoride membrane. The membranes were blocked with 5% nonfat milk at room temperature for 1 h. The primary antibodies were used: Hsp 27 (Santa Cruz; sc-1049; 1:1000), p-Hsp27(Santa Cruz; sc-12359; 1:1000), GAPDH (ab9482; 1:5000 dilution) (Abcam, Cambridge, MA, USA), Oct-3/4 (sc-8630; 1:1000), Nanog (sc-81961; 1:1000), ATP-binding cassette sub-family G member 2 (ABCG2) (sc-8630; 1:1000), MDR-1 (sc-8630; 1:1000), p38MAPK (sc-8630; 1:1000), p-p38MAPK(sc-8630; 1:1000), Cl-capase-3 (sc-8630; 1:1000) and Cl-PARP (sc-81961; 1:1000) in TBST buffer containing 3% nonfat milk at 4°C overnight and subsequently with anti-mouse and rabbit anti-goat secondary antibody conjugated with peroxidase (1:1000) (Santa Cruz Biotechnology) at 25°C for 1 h. The immunoblots were developed using an enhanced chemiluminescence system, and the luminescence was visualized on X-ray film.

### Clinical samples collection

Tissue specimens of 40 pulmonary adenocarcinoma patients with history of cisplatin-treatment were collected and retrieved from the archives of the Department of Pathology, Tri-Service General Hospital, Taipei, Taiwan from 2009 to 2010. There were 18 cases either lost to follow-up or having insufficient clinicopathological data for analysis and were therefore excluded. Among 22 patients with lung cancer in which contained sufficient analyzing tissues or cells then were chosen to be the main study material, it consisted of representative paraffin blocks of tissue section or cell blocks from pleural effusion. Follow-up work to assess the overall survival was conducted for at least 60 months.

### Ethics statement

A total of 40 human samples were obtained for this study. Samples used for analysis in the laboratory were de-identified and not linked with any personal health information. All parts of this study were approved by the Tri-Service General Hospital Institutional Review Board.

### Immunohistochemistry

Tissue sections or cell block were de-waxed in xylene and rehydrated in alcohol. Antigen retrieval was carried out by incubation in 10 mM citrate buffer (pH 6.0) at 95°C for 40 min. Endogenous peroxidase was blocked with 0.3% hydrogen peroxide for 10 min then incubated with 5% normal horse serum in phosphate-buffered saline (PBS) for 60 min at room temperature to block non-specific antibody reaction. After a wash with Tris-buffered saline plus 0.1% Tween 20(TBST), slides were incubated overnight at 4°C with primary antibodies, thyroid transcription factor-1 (TTF-1), Napsin A, and p-Hsp27 (sc-12359; 1:100, Santa Cruz Biotechnology, Inc., CA. USA). After being rinsed in TBST, slides were incubated for 30 min at room temperature with biotinylated secondary antibody followed by streptavidin–biotinylated–enzyme complex (streptABComplexes kit; Dako, Glostrup, Denmark). Subsequently, they were stained with 0.003% 3, 3-diaminobenzidine tetrahydrochloride, counterstained with Mayer's hematoxylin, dehydrated, and mounted.

### Evaluation of immunohistochemical staining results

The histopathological slides of all the specimens were reviewed concurrently and evaluated independently by two well-trained pathologists using the same type of microscope without any prior knowledge of each patient's clinical details. Firstly, all the study cases of pulmonary adenocarcinomas included are initially identified by histology and further confirmed immunohistochemically with reactivity to two common representative biomarkers for primary pulmonary adenocarcinoma, TTF-1 and Napsin A [22]. To precisely and objectively evaluate the intensity of p-Hsp27, we used a Zeiss AxioImager-Z1 microscope to take pictures of all slides and measured the intensity of the marker using Program Metamorph software. The intensities of the immunoreactivity of tumor cells were scored into three standardized categories: 0 (no staining), 1 (weak staining), 2 (moderate staining) and 3 (strongest intensity). To measure the distributions of p-Hsp27, we used Adobe Photoshop CS5 to measure the distribution of the marker as a percentage of the positively stained tumour cells (from 0 to 100) in each section of the total tumour volume. A value of 10% or higher for positively stained tumor cells was considered a positive reaction. To compare the expressions for each case, the percent of positive cells at each level of intensity was multiplied by the corresponding intensity (from 0 to 3) to obtain an immunoreactivity score ranging from 0 to 300 as described in our previous report [23, 24].

### Statistical analysis

The independent Student's t test or ANOVA was used to compare the continuous variables between groups, whereas the X^2^ test was applied for the comparison of dichotomous variable. The level of statistical significance was set at 0.05 for all tests. All statistical analyses were performed using SPSS version 20.0 (SPSS, Inc., Chicago, IL, USA).
